# Effects of psychotherapies for posttraumatic stress disorder on sleep disturbances: Results from a randomized clinical trial

**DOI:** 10.1016/j.brat.2017.07.001

**Published:** 2017-10

**Authors:** Elizabeth Woodward, Ann Hackmann, Jennifer Wild, Nick Grey, David M. Clark, Anke Ehlers

**Affiliations:** a Department of Experimental Psychology, University of Oxford, UK; b National Institute for Health Research (NIHR), Oxford Health Biomedical Research Centre, Oxford, UK; c National Institute for Health Research (NIHR) Mental Health Biomedical Research Centre, South London Maudsley NHS Foundation Trust, King's College London, UK

**Keywords:** Posttraumatic stress disorder, Sleep disturbances, Randomized controlled trial, Cognitive behavioural therapy

## Abstract

The effectiveness and mechanisms of psychotherapies for posttraumatic stress disorder (PTSD) in treating sleep problems is of interest. This study compared the effects of a trauma-focused and a non-trauma-focused psychotherapy on sleep, to investigate whether 1) sleep improves with psychotherapy for PTSD; 2) the degree of sleep improvement depends on whether the intervention is trauma or nontrauma-focused; 3) the memory-updating procedure in cognitive therapy for PTSD (CT-PTSD) is associated with sleep improvements; 4) initial sleep duration affects PTSD treatment outcome; and 5) which symptom changes are associated with sleep duration improvements. Self-reported sleep was assessed during a randomized controlled trial (Ehlers et al., 2014) comparing CT-PTSD (delivered weekly or intensively over 7-days) with emotion-focused supportive therapy, and a waitlist. Sleep duration was reported daily in sleep diaries during intensive CT-PTSD. CT-PTSD led to greater increases in sleep duration (55.2 min) and reductions in insomnia symptoms and nightmares than supportive therapy and the waitlist. In intensive CT-PTSD, sleep duration improved within 7 days, and sleep diaries indicated a 40-min sleep duration increase after updating trauma memories. Initial sleep duration was not related to CT-PTSD treatment outcome when initial PTSD symptom severity was controlled. The results suggest that trauma-focused psychotherapy for PTSD is more effective than nontrauma-focused therapy in improving self-reported sleep, and that CT-PTSD can still be effective in the presence of reduced sleep duration.

## Highlights

• Randomised controlled design comparing psychotherapies for posttraumatic stress disorder (PTSD) on sleep.
• Self-reported sleep improves with cognitive therapy for PTSD (CT-PTSD).
• Sleep improves more with a trauma focused vs. non-trauma focused psychotherapy for PTSD.
• Sleep diaries showed a sleep duration improvement after updating trauma memories in therapy.
• PTSD can be successfully treated in the presence of reduced sleep duration.

## 1 Introduction

Sleep disturbances, such as difficulty falling and staying asleep and nightmares are two of the diagnostic symptoms of posttraumatic stress disorder (PTSD) (American Psychiatric Association, 2013). Sleep problems in PTSD include reduced self-reported and objective sleep duration and lower reported sleep quality (for review see Cox & Olatunji, 2016) and up to 60% of people with PTSD and insomnia complaints also meet criteria for an insomnia disorder (Ohayon & Shapiro, 2000).

Trauma-focused psychological therapies are the first-line recommended treatments for individuals suffering from PTSD, including when comorbid insomnia is present. It is therefore important to understand the effects of trauma-focused PTSD treatments on sleep outcomes in order to maximize PTSD treatment efficacy. Only a small number of studies have investigated the effects of trauma-focused PTSD therapies on sleep outcomes (Belleville et al., 2011, Brownlow et al., 2016, Galovski et al., 2009, Galovski et al., 2016, Gutner et al., 2013, Levrier et al., 2016, Lommen et al., 2015, Nishith et al., 2003, Raboni et al., 2006, Zayfert and DeViva, 2004). Improvement in sleep has been found for prolonged exposure (PE) and cognitive processing therapy (CPT) for PTSD (Brownlow et al., 2016, Galovski et al., 2016, Galovski et al., 2009, Gutner et al., 2013), eye-movement desensitization and reprocessing therapy (Raboni et al., 2006), other cognitive behavioural therapies (Belleville et al., 2011, Levrier et al., 2016, Nishith et al., 2003, Zayfert and DeViva, 2004), and cognitive therapy for PTSD (CT-PTSD) (Lommen et al., 2015). Direct comparisons of two evidence-based, trauma-focused PTSD therapies, CPT and PE, found no differences in sleep improvement between treatments (Galovski et al., 2009; Gutner et al., 2013).

However, studies have also found that despite improvements in self-reported sleep duration and/or quality (Belleville et al., 2011, Galovski et al., 2009, Gutner et al., 2013, Lommen et al., 2015, Raboni et al., 2006, Zayfert and DeViva, 2004), nightmares (Belleville et al., 2011, Gutner et al., 2013, Levrier et al., 2016), and insomnia symptoms (e.g., Gutner et al., 2013), sleep difficulties are commonly residual after PTSD therapy (Belleville et al., 2011, Galovski et al., 2016, Galovski et al., 2009, Gutner et al., 2013, Lommen et al., 2015), including in those who have recovered from PTSD (Zayfert & DeViva, 2004).

It would therefore be important to investigate further which PTSD therapies, and which aspects of PTSD therapy, best promote sleep improvements, and whether sleep problems are residual to the same extent across different psychotherapies for PTSD. To our knowledge, no study has yet compared the effects of trauma and nontrauma-focused psychotherapy for PTSD on sleep, in adults. If trauma-focused therapy has superior effects on sleep compared to nontrauma-focused therapy, this may suggest that the focus on trauma memories and their meaning in these treatments may contribute to sleep improvements. It is also of interest to explore whether sleep improvement coincides with certain procedures in treatment that aim to change the “here and now” quality of trauma memories, such as the *updating memories procedure* in CT-PTSD (Ehlers & Clark, 2000). In this procedure, the individually most upsetting moments in memory are linked to less threatening meanings that the patient and therapist have identified from the course of events (e.g., “I did not die”) or through cognitive restructuring (e.g., “I could not have prevented the trauma even if I had acted differently”).

Furthermore, few studies have investigated which symptom changes are associated with sleep improvements with trauma-focused PTSD therapy (e.g., Lommen et al., 2015). Understanding whether treatment changes symptoms that have been associated with sleep disturbances in PTSD, such as arousal (see Sinha, 2016) and trauma-related nightmares (e.g., Woodward, Arsenault, Murray, & Bliwise, 2000), would be informative.

Finally, research demonstrating the importance of sleep in learning and memory, emotional processing (Diekelmann et al., 2012, Wagner et al., 2006, Walker and van der Helm, 2009, Yoo et al., 2007), and retention and generalization of fear extinction learning (Kleim et al., 2013, Pace-Schott et al., 2012) has contributed to concerns that reduced sleep duration may have a detrimental effect on response to psychological PTSD treatments. Many of the trauma-focused treatments for PTSD involve some form of exposure to trauma memories and reminders (Schnyder et al., 2017), and it is possible that reduced sleep duration may interfere with the effects of exposure through impairing retention of fear extinction learning. Poor sleep may also interfere by impacting an individual's ability to retain learning from the treatment session through reducing concentration and attention in therapy, or interfering with consolidation of new information from the session, such as updated information and meanings in the trauma memory. There is also some evidence that poor sleep quality predicts a slower response to PTSD treatment, in people with PTSD and comorbid major depression (Lommen et al., 2015). However, recent studies have also found that nightmares did not impact the efficiency of CBT for PTSD (Levrier et al., 2016), and that while sleep-directed hypnosis before CPT for PTSD improved sleep more than a control condition, it did not lead to greater improvements in PTSD symptoms after CPT (Galovski et al., 2016), and thus initial evidence is so far inconclusive. Further research is needed into the effects of reduced sleep duration on PTSD treatment outcomes.

### 1.1 Study aims

The primary aim of this study was to compare the effects of a trauma-focused and a nontrauma-focused psychotherapy for PTSD on self-reported sleep, in a randomised controlled trial design, in a secondary analysis of a clinical trial by Ehlers and colleagues (2014). This trial compared trauma-focused CT-PTSD (Ehlers and Clark, 2000, Ehlers et al., 2005) to emotion-focused supportive therapy, an active nontrauma-focused treatment, and a waitlist control. Both psychotherapies (cognitive and supportive therapy) led to greater changes in PTSD symptoms than the waitlist, and CT-PTSD lead to greater improvements in PTSD symptoms than supportive therapy (see Ehlers et al., 2014 for full results). For the sleep study presented here, sleep duration was the primary outcome measure as it was the most regularly available sleep outcome. Additional analyses included sleep quality and insomnia symptoms that were assessed before and after treatment.

Specifically, the present study investigated whether 1) trauma-focused (CT-PTSD) and nontrauma-focused psychological treatment (supportive therapy) lead to greater improvement in sleep than the waitlist control, and 2) the degree of sleep improvement depends on whether the intervention is trauma or nontrauma-focused. If psychotherapy generally improves sleep, both CT-PTSD and supportive therapy would be expected to lead to greater improvement in sleep than a waitlist. If trauma-focused therapy is important for improving sleep with PTSD treatment, then CT-PTSD would be expected to lead to greater improvements in sleep than supportive therapy, which focuses on present feelings and problems, rather than trauma memories and their meanings.

The study further investigated 3) whether the memory-updating procedure in CT-PTSD was associated with sleep improvements. To do this, the study compared two versions of CT-PTSD, a standard weekly version and an intensive version, in which the interventions which promote memory-updating were delivered over five to seven working days, compared to three months for standard CT-PTSD. This enabled investigation of whether sleep improvements coincided with interventions that facilitate memory-updating: First, by comparing standard and intensive treatment at a time point when memory-updating had taken place for the intensive, but not the standard weekly CT-PTSD group; and second, by analysing daily sleep diaries completed by the intensive CT-PTSD group, to compare sleep directly before and after memory-updating. If linking the worst moments in trauma memories with less threatening meanings promotes sleep improvements with CT-PTSD, then it would be expected that sleep would significantly improve after the memory-updating procedure in therapy.

Next, 4) the effect of sleep duration on PTSD treatment outcome was also investigated. If reduced initial sleep duration interferes with treatment outcome, it would be expected that lower sleep duration at pre-treatment would predict more severe PTSD symptoms at post-treatment.

Finally, 5) in patients who received CT-PTSD, associations between improvement in sleep duration and symptoms hypothesised to be related to sleep improvements were explored. These were reductions in nightmares, hyperarousal and anxiety about going to bed.

## 2 Methods

### 2.1 Participants

Participants were out-patients with PTSD (N = 121) who participated in a randomized controlled trial comparing psychological treatments for PTSD and a waitlist condition (Ehlers et al., 2014, ISRCTN: 48524925). The sample comprised 71 women and 50 men (aged 18–65) who met diagnostic criteria for PTSD, as determined by the Structured Clinical Interview for DSM-IV (First, Gibbon, Spitzer, & Williams, 1996). Participants were included if PTSD was their primary diagnosis, and their current intrusive memories were linked to one or two traumas in adulthood. Details of trauma type and demographics are shown in Table 1 (see Ehlers et al., 2014, for further sample details).

**Table 1:** Demographic information and trauma characteristics by treatment condition.

Demographics		Intensive Cognitive Therapy (*N* = 30)				Standard Cognitive Therapy (*N* = 31)				Supportive Therapy (*N* = 30)				Waitlist (*N* = 30)			
		M	SD	N	%	M	SD	N	%	M	SD	N	%	M	SD	N	%
Sex	Female			18	60.0			18	58.1			17	56.7			18	60
	Male			12	40.0			13	43.9			13	43.4			12	40
Ethnic Group	Caucasian			22	73.3			20	64.5			22	73.3			21	70
	Ethnic Minority			8	26.7			11	35.5			8	26.7			9	30
Age	(Years)	39.7	12.4			41.5	11.7			37.8	9.9			36.8	10.5		
**Traumas**		M	SD	N	%	M	SD	N	%	M	SD	N	%	M	SD	N	%
Type of Main Traumatic Event	Interpersonal Violence^1^			12	40.0			12	38.7			11	36.7			10	33.3
	Accidents/Disaster			11	36.7			11	22.6			14	10.0			10	33.3
	Death/Harm to Others			2	6.7			1	3.2			2	6.7			4	13.3
	Other			5	16.7			7	22.6			3	10.0			6	20
Time since Main Traumatic Event	3 months - 1 year			10	33.3			14	45.2			8	27.8			14	46.7
	1 to 2 years			10	33.3			5	16.1			7	24.1			6	20
	2 to 4 years			7	23.3			11	35.5			8	27.6			3	10
	More than 4 years			3	10.0			1	3.2			6	20.7			7	23.3
History of Other Trauma	Yes			22	63.3			21	67.7			23	76.7			20	66.7
	No			8	26.7			10	32.3			7	23.3			10	33.3
Reported History of Childhood Abuse	Yes			5	16.7			2	6.5			4	13.3			3	10.0
	No			25	83.3			29	93.5			26	86.7			27	90.0
**Comorbidity**																	
Anxiety Disorder	Yes			10	33.3			7	22.6			10	33.3			10	33.3
	No			20	66.7			24	77.4			20	66.7			20	66.7
Depressive Disorder	Yes			12	40.0			7	22.6			11	36.7			14	46.7
	No			18	60.0			24	77.4			19	63.3			16	53.3
Substance Abuse	Yes			6	20.1			6	19.5			6	20.1			2	6.7
	No			24	80.0			25	80.6			24	80.0			28	93.3
History of Substance Dependence	Yes			2	6.7			4	12.9			2	6.7			1	3.3
	No			28	93.3			27	87.1			28	93.3			29	96.7
Axis II disorder	Yes			7	23.3			5	16.1			4	13.3			8	26.7
	No			23	76.7			26	83.9			26	86.7			22	73.3

### 2.2 Treatment conditions

Participants were randomly allocated to one of four conditions: (1) trauma-focused cognitive therapy for PTSD (CT-PTSD), delivered over 12 weeks (N = 31); (2) intensive CT-PTSD (N = 30) delivered over five to seven days; (3) emotion focused supportive therapy, delivered over 12 weeks (N = 30); or (4) a 14-week waitlist (N = 30). By the 14-week assessment patients in all treatment conditions had received equivalent therapist input of up to 20 hours of therapy. The therapy conditions are outlined below (see Ehlers et al., 2014, for full details).

#### 2.2.1 Cognitive therapy for PTSD (CT-PTSD; Ehlers et al., 2005)

This is a trauma-focused therapy, which follows Ehlers and Clark's model of PTSD (Ehlers & Clark, 2000). The treatment aims to reduce the patient's sense of current threat by changing problematic meanings of the trauma and its consequences, elaborating and updating the memories of the trauma with information that gives them a less threatening meaning at present, discriminating triggers of intrusive memories, and changing behaviours and cognitive processes that maintain PTSD, such as rumination and safety behaviours. For details of procedures see: http://oxcadat.psy.ox.ac.uk/downloads/CT-PTSD%20Treatment%20Procedures.pdf/view. Sleep was not directly targeted. CT-PTSD was delivered in 12 weekly sessions over three months (standard CT) or as an intensive therapy (intensive CT), which was delivered daily over five to seven working days and two shorter sessions one week and one month later.

#### 2.2.2 Emotion-focused supportive therapy (EST)

This non-directive treatment focuses on the patient's emotions rather than cognitions. After normalizing PTSD symptoms, the therapist gave the rationale that the trauma had left the patient with unprocessed emotions and that therapy would provide them with support and a safe context to address their unresolved emotions. Patients could freely choose what problems to discuss in the session, including any aspect of the trauma. Therapists helped patients clarify their emotions and solve problems. EST was delivered in 12 weekly sessions over 3 months.

#### 2.2.3 14 week waitlist

Patients waited for 14 weeks before random allocation to either weekly (*n* = 13) or intensive CT-PTSD (*n* = 11).

### 2.3 Measures

Self-reported PTSD symptom severity (excluding sleep) and sleep duration were assessed at baseline (pre-treatment/wait), 6 weeks and 14 weeks (post-treatment/wait). Treatment groups were also assessed at 3 weeks, and at the 27- and 40-week follow-ups. Assessments with an independent rater unaware of allocation were conducted at baseline, 14 weeks, 27 and 40 weeks. Other sleep outcomes (insomnia symptoms, nightmares and sleep quality) were assessed at baseline and 14 weeks. Sleep quality and sleep duration (total time spent asleep) are two of a number of sleep outcomes recommended in the subjective assessment of sleep (Buysse, Ancoli-Israel, Edinger, Lichstein, & Morin, 2006), and provide information about the subjective experience of sleep.

#### 2.3.1 Main measures completed by all groups

Patients in the treatment groups completed a PTSD symptom measure and reported sleep duration at baseline, 3, 6, 14, 27 and 40 weeks, and those in the waitlist group at baseline and weeks 6 and 14.

##### 2.3.1.1 PTSD symptoms

The main PTSD symptom measure for the purposes of the present study was the Posttraumatic Diagnostic Scale (PDS) (Foa, Cashman, Jaycox, & Perry, 1997), a self-report questionnaire measuring the overall severity of 17 PTSD symptoms specified in the DSM-IV. The PDS has shown good reliability and concurrent validity with other PTSD measures. Sum scores range from 0 to 51. In the present study, the results of independent assessments with the Clinician-administered PTSD Scale (CAPS) for DSM-IV (Blake et al., 1995) showed identical results to the PDS for outcome, but the CAPS was given less frequently than the PDS (see Ehlers et al., 2014), and so PDS-assessed PTSD symptom severity is reported. The PDS sleep item (‘*having trouble falling or staying asleep*’), was omitted for the purposes of the present analyses, and the PTSD severity score was based on the remaining 16 items.

##### 2.3.1.2 Self-reported sleep duration

Patients reported how many hours of sleep they had had on average per night over the past week. This was used as the primary sleep outcome measure in this study, as duration was available at every assessment point, and in the sleep diary (see below), whereas the additional sleep measures (with the exception of the PDS insomnia item) were only available at initial assessment and 14-weeks.

#### 2.3.2 Additional measures completed by all groups

To explore other aspects of sleep and potentially associated processes, further measures were taken at baseline and 14 weeks.

##### 2.3.2.1 Self-reported sleep quality

Patients rated their overall sleep quality over the past week (‘*overall, how well did you sleep*?’ rated on a scale from 0 *'not at all well*' to 100 *'very well*'). Two participants in each group had missing data because the measure was introduced with delay.

##### 2.3.2.2 Insomnia symptoms

Severity of sleep problems was also assessed with the CAPS insomnia item (*‘problems falling or staying asleep’;* frequency plus intensity rating, each measured on a scale from 0 to 4), at initial assessment and 14 weeks. For comparison, we also analysed the PDS insomnia item 13 (*‘having trouble falling or staying asleep’*; frequency rated on a scale from 0 ‘*not at all*’ to 3 ‘*5 or more times a week*’) at these time points. The percentage of patients meeting the clinical cut-off for the CAPS insomnia item (frequency at least 1, and intensity at least 2 *’30 – 90 min loss of sleep’*) at initial assessment and 14 weeks, was also calculated.

##### 2.3.2.3 Associated symptoms: arousal, nightmares and pre-bed anxiety

Symptoms that may be associated with treatment effects on sleep duration included nightmares (CAPS and PDS item), hyperarousal symptoms, excluding difficulty falling and staying asleep (mean of CAPS hyperarousal items, and of PDS hyperarousal items: 14, 15, 16 and 17), and patient ratings of feeling anxious about going to bed (on a scale from 0 *'not at all*' to 100 *'very much'*).

#### 2.3.3 Measures completed by intensive cognitive therapy patients only

##### 2.3.3.1 Sleep diary (daily reported sleep duration)

Participants receiving intensive CT-PTSD (including those initially allocated to waitlist; N = 32) reported their sleep duration in a sleep diary each morning for 6 weeks in total; two weeks pre-treatment, two weeks during the intensive treatment phase (one week) and the following week (one week), and two weeks before the 6-week assessment. Participants reported how many hours and minutes they had slept the previous night. Weekly self-reported sleep duration (from the questionnaires) correlated with the mean sleep duration reported in the diary at pre-treatment (*r* = 0.85, N = 32), and post-treatment (*r* = 0.78, *n* = 26).

### 2.4 Data analysis

Analyses were conducted using SPSS version 22. To test whether psychological treatments lead to improvements in sleep (Question 1), an analysis of covariance (ANCOVA) compared reported sleep *duration* at 6 and 14 weeks, with the between-subject factor ‘condition’ (CT, EST, wait) and the within-subject factor ‘time’, and the covariate baseline sleep duration. Overall effects of condition are reported, as well as adjusted mean differences and 95% confidence intervals (CI) for differences between the treatment conditions and waitlist. Effect sizes (ES) are reported as partial η2, with small ES ≥ 0.01; medium ≥ 0.06; and large ≥ 0.14. Additional ANCOVAs compared the other sleep measures taken at baseline and 14 weeks (sleep quality, insomnia items from the CAPS and PDS) with between factor ‘condition’ (CT, EST, wait), controlling for baseline severity. Changes in the proportion of patients meeting the clinical cut-off on the CAPS insomnia item before and after therapy were compared with related-samples McNemar tests.

To test whether cognitive therapy leads to greater improvement in sleep than supportive therapy (Question 2), an ANCOVA compared reported sleep *duration* at 6, 14, 27 and 40 weeks, with the between factor ‘condition’ (CT, EST) and the within-subject factor ‘time’, controlling for baseline sleep duration. For this analysis, the treatment groups (CT, EST) included participants that were initially allocated to the waitlist. Treatment group differences on additional sleep measures (sleep quality, insomnia symptoms; CAPS and PDS) measured at 14 weeks were analysed with further ANCOVAs, controlling for baseline severity.

For comparison, parallel analyses are reported for PTSD severity excluding sleep (PDS, see Ehlers et al., 2014, for comprehensive analyses of PTSD and other outcome measures).

Question 3 was analysed in three steps. First, an ANCOVA compared sleep duration in intensive and standard CT at 3 weeks, controlling for baseline sleep duration. By the 3-week assessment, the intensive, but not the standard CT group, had completed the memory-updating interventions. This analysis included the patients randomly allocated after waiting for treatment (N = 44 standard CT, N = 41 intensive CT). Second, sleep diaries completed by the intensive CT group were analysed. A one-way repeated measures ANOVA, and planned contrasts compared mean reported sleep duration before, during and after intensive CT. Third, the impact of the memory-updating procedure on sleep duration was examined by comparing sleep duration reported in the diary before and after the first discrete period of memory-updating in intensive CT, with sleep duration before and an equivalent amount of time after a randomly selected pre-treatment day with no intervention (Mean day = 4.78, SD = 2.75). Memory-updating was most commonly done for the first time on the second day of intensive treatment (39.3% of patients). Updating was done between one and four times (M = 2.09, SD = 1.04), and on average concluded on day 4 of treatment (M = 4.12, SD = 1.54).

To address Question 4 (impact of reduced sleep duration on PTSD treatment outcome), correlation and regression analyses examined the relationship between sleep duration at initial assessment and end of treatment (14 weeks), and treatment outcome (PDS at 14 and 40 weeks), controlling for PDS. This analysis included all 85 patients who received cognitive therapy, including those who had initially been allocated to the waitlist. An ANCOVA checked beforehand that there were no effects of type of CT (intensive versus standard) or allocation (immediate versus post-wait), nor an interaction, all *p*'s > 0.32.

To investigate other measures that may be associated with improvement in sleep duration in therapy, symptoms that were hypothesised may be associated with sleep improvements were explored (Question 5). ANCOVAs tested differences between conditions (CT, EST, wait) for nightmares, hyperarousal symptoms (CAPS, PDS), and patient-rated anxiety about going to bed at 14 weeks, controlling for baseline severity. In patients treated with CT-PTSD, sleep duration change with treatment was correlated with change in nightmares and hyperarousal symptoms (CAPS, PDS), and anxiety about going to bed.

## 3 Results

### 3.1 Question 1: Do psychological therapies for PTSD improve self-reported sleep?

#### 3.1.1 Sleep duration

**Fig. 1:**
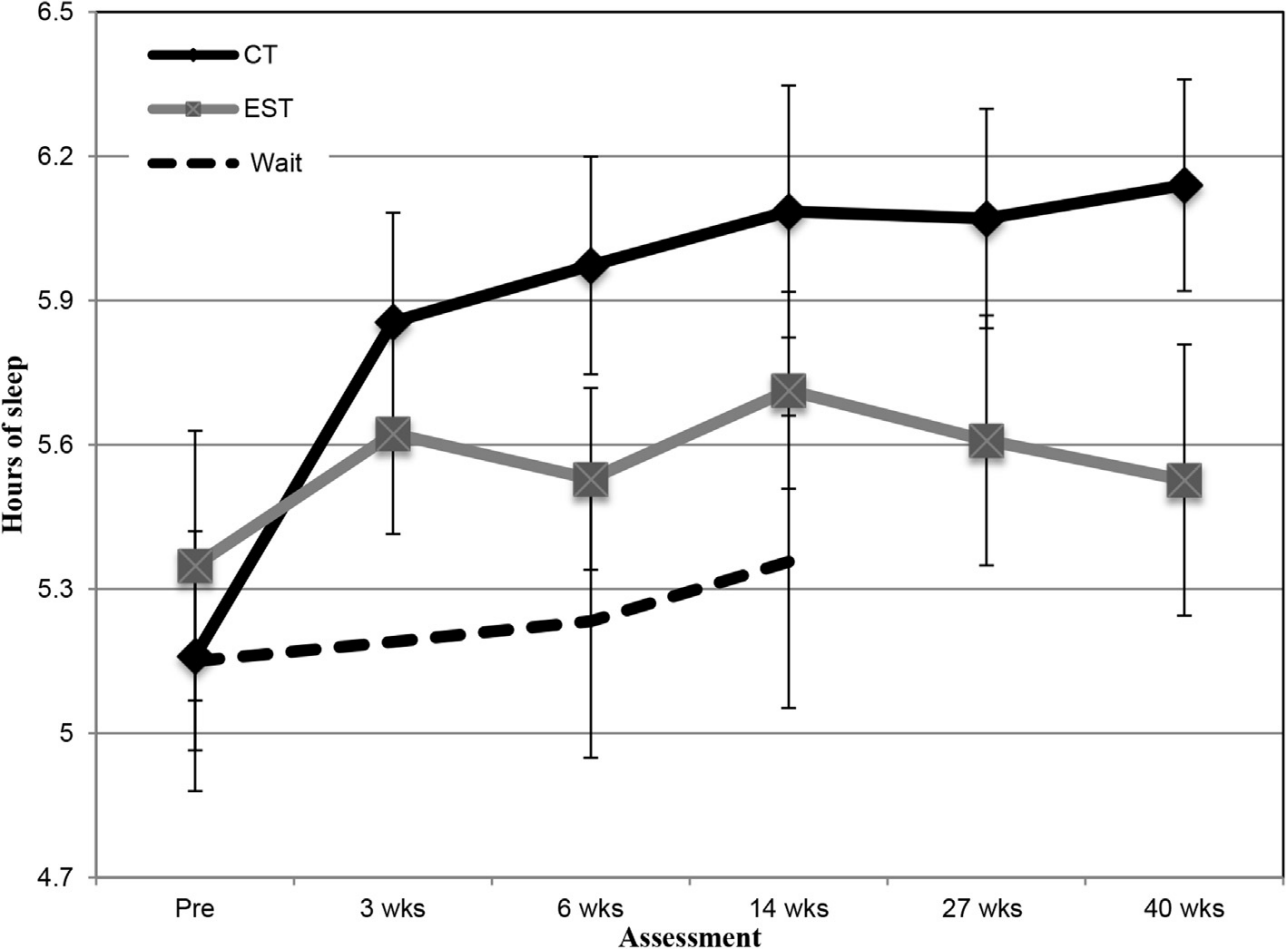
Mean hours of sleep (and standard error bars) reported by patients receiving cognitive therapy (CT; standard and intensive combined, immediate allocations only), emotion-focused supportive therapy (EST) and waitlist.

Fig. 1 illustrates the changes in reported sleep duration across the assessment points for cognitive therapy, supportive therapy and waitlist (see Table 2 for descriptive statistics). The ANCOVA showed a significant difference between the conditions, *F*(2, 117) = 4.30, *p* = 0.016, partial η^2^ = 0.07. Cognitive therapy, but not supportive therapy, led to greater improvement in sleep duration than the waitlist control, baseline-adjusted differences (sleep duration) for CT = 0.73 (95% CI = 0.20 to 1.26), *p* = 0.008; and for EST = 0.21 (95% CI = −0.41 to 0.82), *p* = 0.51. Patients receiving CT reported sleeping 55.2 min longer after treatment (14 weeks), compared to 21.6 min longer with supportive therapy and 12.6 min on the waitlist (see Table 2).

**Table 2:** Mean and standard deviations (SD) at each assessment point for the psychological treatment and waitlist conditions, and for intensive and standard cognitive therapy (including those initially allocated to the waitlist).

	Cognitive Therapy (CT) (N = 61)		Supportive Therapy (N = 30)		Waitlist (N = 30)		Intensive CT, including post-wait (N = 41)		Standard CT, including post-wait (N = 44)	
Hours of Sleep										
	Mean	SD	Mean	SD	Mean	SD	Mean	SD	Mean	SD
Baseline	5.16	1.53	5.35	1.54	5.15	1.48	5.13	1.53	5.38	1.69
3 weeks	5.86	1.77	5.62	1.14	–	–	6.11	1.59	5.62	1.84
6 weeks	5.97	1.77	5.53	1.03	5.23	1.56	5.95	1.86	5.98	1.66
14 weeks	6.08	2.05	5.71	1.12	5.36	1.66	6.03	1.66	6.31	2.23
27 weeks	6.07	1.78	5.61	1.42	–	–	5.88	1.72	6.42	1.71
40 weeks	6.14	1.72	5.53	1.54	–	–	6.08	1.78	6.51	1.85
Sleep Diary Data: Hours of Sleep										
Pre-treat	–	–	–	–	–	–	5.43	1.44	–	–
During treat	–	–	–	–	–	–	5.62	1.34	–	–
Post-treat	–	–	–	–	–	–	6.43	1.73	–	–
PTSD Severity (excluding sleep)										
Baseline	30.69	6.79	32.11	6.93	30.05	7.33	30.69	6.94	29.74	7.01
3 weeks	18.59	10.27	26.43	11.48	–	–	16.21	8.90	22.67	10.51
6 weeks	14.53	9.56	21.73	12.33	29.76	6.86	13.86	8.41	16.80	11.71
14 weeks	9.75	9.51	18.40	13.03	27.04	9.02	10.77	8.60	8.73	9.66
27 weeks	11.01	11.01	17.37	12.06	–	–	11.82	10.01	8.81	10.31
40 weeks	10.45	10.80	19.38	13.96	–	–	12.17	10.98	9.56	10.35

Further measures at Baseline and 14 Weeks										
Sleep Quality										
Baseline	34.82	21.30	31.07	20.61	36.07	19.69	35.85	20.25	33.08	20.64
14 weeks	58.95	27.33	46.61	26.81	33.04	16.74	57.56	31.03	56.79	23.60
Insomnia item (PDS)										
Baseline	1.98	1.01	2.15	0.88	2.40	0.72	1.98	0.98	2.17	0.92
14 weeks	0.89	1.05	1.57	0.97	2.20	0.85	0.80	1.18	0.99	0.99
Insomnia item (CAPS)										
Baseline	5.48	2.43	6.05	1.83	5.08	2.22	5.11	2.38	5.40	2.62
14 weeks	2.99	3.23	4.50	3.33	4.73	2.66	2.63	3.27	3.01	3.04
Insomnia item (CAPS, percent and N meeting clinical cut-off)										
Baseline	78.7%	48/61	90.0%	27/30	76.7%	23/30	78.0%	32/41	75.0%	33/44
14 weeks	44.3%	27/61	63.3%	19/30	70.0%	21/30	46.3%	19/41	36.4%	16/44
Bad dreams and nightmares (PDS)										
Baseline	1.57	0.96	1.53	0.86	1.53	0.94	1.43	0.93	1.51	1.05
14 weeks	0.43	0.67	0.90	0.99	1.17	0.96	0.42	0.72	0.46	0.74
Bad dreams and nightmares (CAPS)										
Baseline	3.75	2.60	3.42	2.61	3.27	2.70	3.33	2.62	3.82	2.63
14 weeks	1.31	2.13	2.27	2.72	3.17	2.62	1.27	2.14	1.29	2.12
Hyperarousal (excluding sleep, PDS)										
Baseline	2.09	0.58	2.15	0.54	2.05	0.60	2.05	0.60	2.14	0.53
14 weeks	0.80	0.74	1.32	0.86	2.00	0.74	0.74	0.75	0.93	0.68
Hyperarousal (excluding sleep, CAPS)										
Baseline	18.29	5.16	18.55	4.62	17.00	5.74	17.47	4.18	19.18	5.97
14 weeks	8.26	7.69	12.28	7.34	17.43	6.28	7.69	7.75	9.49	7.48
Anxiety going to bed										
Baseline	42.54	30.59	33.67	27.98	46.33	26.45	39.43	32.46	41.71	26.45
14 weeks	20.98	30.04	35.17	31.83	38.50	29.04	17.72	25.60	22.56	30.23

#### 3.1.2 PTSD symptom severity (excluding sleep)

PTSD symptom changes are reported for comparison. Table 2 shows the changes in self-reported PTSD symptoms (excluding sleep). The ANCOVA showed a significant difference between the conditions, *F*(2, 117) = 40.40, *p* < 0.001, partial η^2^ = 0.41. Both treatments led to greater reductions in PTSD symptoms compared to waitlist; baseline-adjusted differences for CT = - 16.63 (95% CI = −12.95 to −20.31); EST = −9.55 (95% CI = −5.26 to −13.83).

#### 3.1.3 Additional sleep measures

For self-reported sleep quality, the ANCOVA showed a significant difference between the conditions for self-reported sleep-quality, *F*(2, 109) = 12.77, *p* < 0.001, partial η^2^ = 0.19. Cognitive therapy led to greater changes in sleep quality than waitlist: baseline-adjusted mean difference = 26.54 (95% CI = 16.11 to 36.98), *p* < 0.001, as did supportive therapy baseline-adjusted mean difference = 16.10 (95% CI = 3.97 to 22.23), *p* = 0.01.

For insomnia symptoms assessed with the PDS and CAPS, the ANCOVAs showed significant differences between the conditions: PDS insomnia, *F*(2, 117) = 15.84, *p* < 0.001, partial η^2^ = 0.213; CAPS insomnia, *F*(2, 117) = 5.53, *p* = 0.005, partial η^2^ = 0.09. Cognitive therapy led to greater changes in insomnia than waitlist: baseline-adjusted mean difference for PDS insomnia = −1.12 (95% CI = −1.52 to −0.72), *p* < 0.001; CAPS insomnia = −2.00 (95% CI = −3.23 to −0.77), *p* = 0.002. In contrast, supportive therapy was not superior to the waitlist on CAPS insomnia severity, baseline adjusted mean difference = −0.86 (95% CI = −2.30 to 0.58), *p* = 0.24, but showed advantages over the waitlist on PDS insomnia severity = −0.52 (95% CI = −0.98 to −0.60), *p* = 0.03.

Related-samples McNemar tests showed that with cognitive therapy, *p* < 0.001, and supportive therapy, *p* = 0.021, there was a reduction in the proportion of patients with clinically significant insomnia symptoms between the initial and 14-week assessments, but not in the waitlist group, *p* = 0.69. At the 14-week assessment, 44.3% of patients who had received cognitive therapy and 63.3% of those who had received supportive therapy continued to report clinically significant insomnia symptoms, according to the CAPS.

### 3.2 Question 2: Does the degree of sleep change depend on whether the intervention is trauma or non-trauma-focused?

#### 3.2.1 Sleep duration

The ANCOVA across all assessment points showed a significant effect of treatment condition *F*(1, 112) = 6.50, *p* = 0.012, partial η^2^ = 0.06. Cognitive therapy led to greater improvement in sleep duration than supportive therapy, baseline-adjusted mean difference = 0.60 (95% CI = 0.13 to 1.06) (see Fig. 1 and Table 2).

#### 3.2.2 PTSD symptom severity (excluding sleep)

The ANCOVA for PTSD symptoms without sleep showed a significant difference between the conditions, *F*(1, 112) = 11.99, *p* < 0.001, partial η^2^ = 0.10. Cognitive therapy led to greater improvement in PTSD symptoms than supportive therapy, baseline-adjusted mean difference = −6.54 (95% CI = −2.80 to −10.27).

#### 3.2.3 Additional sleep measures

For sleep quality, there was a trend for cognitive therapy to be superior to supportive therapy: baseline-adjusted mean difference = 8.72 (95% CI = −1.35 to 18.79), *p* = 0.089. Cognitive therapy was superior to supportive therapy on PDS insomnia severity = −0.64 (95% CI = −1.07 to −0.25), *p* = 0.001, and for CAPS insomnia severity = −1.22 (95% CI = −2.40 to −0.04), *p* = 0.043.

### 3.3 Question 3: Does the memory-updating procedure in CT-PTSD facilitate sleep improvements?

#### 3.3.1 Comparison of intensive and weekly cognitive therapy at 3 weeks

##### 3.3.1.1 Sleep duration

The ANCOVA showed a significant effect of group (see Table 2), *F*(1, 82) = 5.75, *p* = 0.019, partial η^2^ = 0.07 on sleep duration at week 3. Patients receiving intensive cognitive therapy, who had completed the updating memory-updating intervention at this stage, reported sleeping longer than those receiving standard CT, who had not yet updated their memories, baseline-adjusted mean difference = 0.67 (95% CI = 0.11 to 1.22).

##### 3.3.1.2 PTSD symptom severity (excluding sleep)

The ANCOVA showed a significant effect of group (see Table 2), *F*(1, 82) = 11.65, *p* = .001, partial η^2^ = .124. Patients receiving intensive therapy reported fewer PTSD symptoms at 3 weeks, than those receiving standard cognitive therapy, baseline-adjusted mean difference = -6.93 (95% CI = -2.89 to -10.97).

#### 3.3.2 Analysis of sleep diaries in intensive cognitive therapy: before and after memory updating intervention

**Fig. 2:**
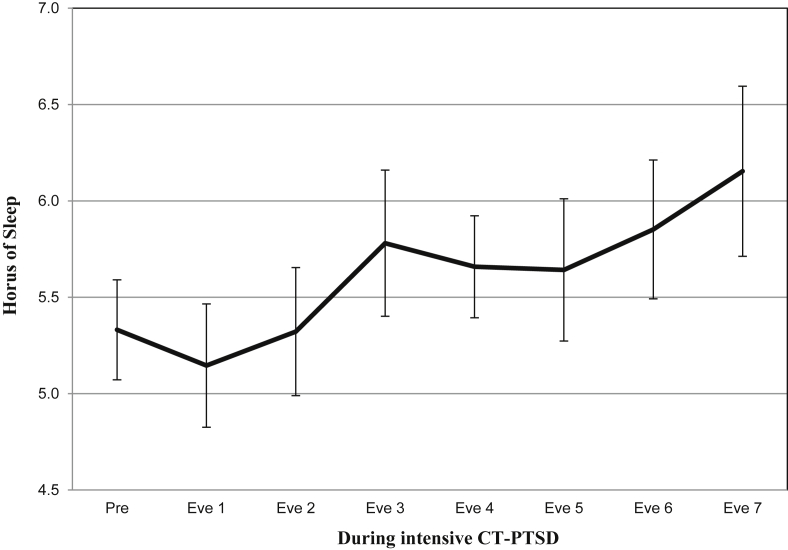
Sleep diary data: Mean sleep duration (with standard error bars) during the 7- day treatment phase of intensive Cognitive Therapy for PTSD. *Pre* = *the mean sleep duration reported in the pre-treatment sleep diary given for comparison. Eve* = *sleep duration that night (Eve 1 would be evening 1 of day 1 of intensive therapy).*

Table 2 shows the mean sleep duration for pre, during and post intensive cognitive therapy as assessed with the sleep diary. The ANOVA showed a significant effect of time on self-reported sleep duration, *F*(2, 44) = 8.68, *p* = 0.001, partial η^2^ = 0.28. There was a significant linear trend across the three time points *F*(1, 22) = 10.27, *p* = 0.004. Planned contrasts showed significant differences in sleep duration between the pre and post-treatment sleep diaries, *F*(1, 22) = 10.27, *p* = 0.004, partial η^2^ = 0.32; and between during-treatment and post-treatment diaries, *F*(1, 23) = 7.52, *p* = 0.01, partial η^2^ = 0.25. There was also a trend for a difference between pre and during-treatment sleep diaries, *F*(1, 28) = 4.13, *p* = 0.05, partial η^2^ = 0.13. Parallel to the results of the questionnaire reported sleep duration, patients receiving intensive therapy reported in the diary an increase in sleep duration of about an hour with therapy (see Table 2 and Fig. 2).

Table 3 shows the comparison of sleep duration reported for the nights before and after memory-updating in therapy. There was a significant increase of 41.04 min of self-reported sleep duration from the night before updating to after updating, *t*(23) = −2.46, *p* = 0.022, partial η^2^ = 0.21, whereas there was no significant change in sleep duration from the night before a randomly selected pre-treatment day to an equivalent period afterwards (with no intervention), *t*(23) = −0.270, *p* = 0.79, partial η^2^ = 0.003.

**Table 3:** Mean sleep duration (hours) and standard deviations before and after memory-updating during intensive Cognitive Therapy for PTSD, and before and after no updating (or intervention).

	Updating (*n* = 24)		No Updating (*n* = 24)	
	Mean	SD	Mean	SD
Pre updating	5.14	1.96	5.52	1.95
Post updating	5.82	2.27	5.40	1.73
Change (in hours)	0.68***	1.36	−0.12	2.23

Note. *** = *p* < 0.001.

### 3.4 Question 4: What is the effect of sleep duration on PTSD treatment outcome?

For patients receiving CT-PTSD, shorter sleep duration at baseline correlated with higher PTSD symptom severity (PDS excluding sleep) at baseline, *r* = −0.48 *p* < 0.001; post-treatment (14 weeks), *r* = −0.23, *p* = 0.007; and at 40-week follow-up, *r* = −0.24, *p* = 0.03. However, regression analyses showed that shorter sleep duration at baseline did not predict PTSD severity at 14 weeks, *ß* = −0.12, *p* = 0.30, and 40 weeks, *ß* = −0.07, *p* = 0.55, over and above what could be predicted from initial PDS scores (excluding sleep). Similarly, poor sleep at the end of treatment correlated with PTSD severity (PDS excluding sleep) at 40-week follow-up, *r* = −0.31, *p* = 0.003, but regression analysis showed that it did not predict PTSD symptoms over and above PTSD severity at the end of treatment, *ß* = −0.07, *p* = 0.32. This shows that sleep duration at baseline and end of treatment did not predict PTSD treatment outcome over and above what could be predicted by PTSD symptom severity.

Change in sleep duration from baseline to end of treatment, however, predicted PTSD severity at the end of treatment over and above initial PTSD symptom severity, *ß* = −0.22, *p* = 0.031.

### 3.5 Question 5: Symptoms that may be associated with sleep improvement

The ANCOVAs showed significant differences at 14 weeks between conditions for: PDS nightmares, *F*(2, 117) = 11.09, *p* < 0.001, partial η^2^ = 0.159; CAPS nightmares, *F*(2, 117) = 10.22, *p* < 0.001, partial η^2^ = 0.149; PDS arousal symptoms (excluding sleep), *F*(2, 117) = 31.79, *p* < 0.001, partial η^2^ = 0.35; CAPS arousal symptoms (excluding sleep), *F*(2, 117) = 21.41, *p* < 0.001, partial η^2^ = 0.27; and anxiety about going to bed, *F*(2, 117) = 5.17, *p* = 0.007, partial η^2^ = 0.081. Cognitive therapy led to larger improvements than the waitlist on each of these measures: baseline-adjusted mean difference for PDS nightmares = −0.75 (95% CI = −1.08 to −0.42), *p* < 0.001; CAPS nightmares = −2.07 (95% CI = −3.00 to −1.15), *p* < 0.001; PDS arousal symptoms (excluding sleep) = - 1.23 (95% CI = −1.53 to −0.92), *p* < 0.001; CAPS arousal symptoms (excluding sleep) = −9.91 (95% CI = −12.92 to −6.90), *p* < 0.001; anxiety about going to bed = −16.19 (95% CI = −28.85 to −3.53, *p* < 0.001. In contrast, supportive therapy was only superior to the waitlist on arousal, PDS arousal = −0.74 (95% CI = −1.09 to −0.39), *p* < 0.001; CAPS arousal = −5.96 (95% CI = −9.44 to −2.48), *p* < 0.001. Cognitive therapy was superior to supportive therapy on all measures: PDS nightmares = −0.48 (95% CI = −0.81 to −0.15), *p* = 0.005; CAPS nightmares = −1.11 (95% CI = −2.02 to −0.178), *p* = 0.02; PDS arousal symptoms (excluding sleep) = - 0.48 (95% CI = −0.79 to −0.18), *p* = 0.002; CAPS arousal symptoms (excluding sleep) = −3.95 (95% CI = −6.94 to −0.96), *p* = 0.010; anxiety about going to bed = −17.29 (95% CI = −30.03; −4.55), *p* = 0.008.

For participants treated with cognitive therapy (N = 85), change in the frequency of nightmares correlated with changes in sleep duration; PDS nightmares *r* = 0.32, *p* = 0.003; CAPS nightmares, *r* = 0.26, *p* = 0.018, as did change in hyperarousal symptoms (excluding sleep); PDS arousal *r* = 0.21, *p* = 0.056, CAPS arousal *r* = 0.388, p < 0.001, and change in anxiety about going to bed, *r* = 0.345, p = 0.001. This shows that sleep duration improvements were correlated with improvements in nightmares, arousal and anxiety about going to bed.

## 4 Discussion

The present study investigated changes in self-reported sleep with psychological treatments for PTSD. Specifically, it was investigated whether 1) psychological therapies for PTSD lead to an improvement in sleep compared to a waitlist condition; 2) the improvement in sleep depends on whether the intervention is trauma or non-trauma-focused; 3) improvements in sleep are related to memory-updating interventions in CT-PTSD; 4) initial sleep duration affects PTSD treatment outcome; and explored 5) whether improvements in hyperarousal, nightmares and anxiety about going to bed are associated with sleep duration improvements.

The results showed that CT-PTSD, but not supportive therapy, led to greater improvement in self-reported sleep duration in comparison to a waitlist control group (Question 1). Among the additional sleep measures, the same pattern was observed for assessor-rated insomnia symptoms (CAPS). For sleep quality and self-reported insomnia symptoms (PDS) both CT-PTSD and supportive therapy were superior to the waitlist. Direct comparisons between the two treatment conditions (Question 2) showed that cognitive therapy lead to greater improvement than supportive therapy in sleep duration and insomnia symptoms (CAPS, PDS), with a trend for superiority on sleep quality. This suggests that trauma-focused therapy is more effective than non-trauma-focused therapy for improving insomnia symptoms in PTSD. Sleep duration increased by nearly one hour on average with CT-PTSD, which focuses on re-processing threatening meanings of the trauma and updating trauma memories with the revised meanings, compared to around 20 minutes with supportive therapy, which was nontrauma-focused. More people had residual insomnia on the CAPS after supportive therapy, than after CT-PTSD. The pattern of results suggests that the sleep changes with CT-PTSD were specific effects of the intervention, rather than effects of the passage of time or non-specific effects of therapy. This conclusion is further supported by the finding that for patients receiving intensive CT, the improvement in sleep duration had already occurred by the 3-week assessment, and that sleep diaries showed a significant increase in sleep duration during the week of intensive treatment (Question 3). These findings suggest that sleep improves with trauma-focused CT, where trauma memories are linked with updating information. This is consistent with a recent paper showing that sleep duration improved by an average of one hour with weekly CT-PTSD in a large sample of PTSD patients in a routine clinical service (Lommen et al., 2015), and with studies showing an improvement in sleep duration after trauma-focused therapies; cognitive processing therapy and prolonged exposure therapy (Galovski et al., 2009, Gutner et al., 2013). The reported gain of one hour of sleep in CT-PTSD is similar to that observed in successful insomnia treatment with insomnia patients (see Mitchell, Gehrman, Perlis, & Umscheid, 2012), which is encouraging. Sleep quality and insomnia improvements with trauma-focused treatment in the present study are consistent with previous studies showing that CBT for PTSD improves insomnia symptoms in PTSD patients (e.g., Gutner et al., 2013). However, by post-treatment (14 weeks), patients’ reported sleep duration was only six hours on average, which is lower than the recommended 7–9 h for adults (see Hirshkowitz et al., 2015). Furthermore, 43.3% of patients were assessed as continuing to experience clinically significant insomnia symptoms (CAPS) after CT-PTSD. This is consistent with the findings of previous studies that insomnia is commonly residual after CBT for PTSD (Belleville et al., 2011, Zayfert and DeViva, 2004). Overall these findings suggest that self-reported sleep duration, quality and insomnia symptoms (difficulties falling and staying asleep) are improved with trauma-focused CT for PTSD, but that insomnia remains residual for up to half of patients after therapy.

The design of the present study allowed for a closer examination of a core procedure (memory-updating) in CT-PTSD that may be important for facilitating sleep duration improvements with PTSD therapy (Question 3). Patients in the intensive CT group reported around a 40-min increase in sleep duration after memory-updating in therapy, whereas there was no change in sleep duration after an equivalent recording period before therapy, where no intervention was done. This suggests that memory-updating may be associated with the sleep duration improvements seen with CT-PTSD. This finding is in line with a previous study that found an increase in objective sleep duration after processing trauma memories with written narrative exposure (Kobayashi, Mellman, Altaee, Howell, & Lavela, 2016). However, in order to conclude that the memory updating intervention in CT-PTSD facilitated sleep improvements, memory updating would need to be compared to an alternative active intervention in CT-PTSD, carried out at the same point in therapy, but that did not include memory-updating. Future studies could investigate this.

The study found that initial sleep duration was not an independent predictor of PTSD treatment response over and above PTSD symptom severity (Question 4), suggesting that reduced sleep duration may not interfere with PTSD treatment outcome. This is consistent with Lommen and colleagues’ (2015) recent study, which found that reduced sleep duration did not interfere with the overall efficacy of CT-PTSD. Concerns for the effects of sleep disturbances in PTSD treatment may come in part from experimental studies with healthy controls, where variables of interest were often measured after full sleep deprivation rather than sleep disturbance or reduction. The results of the current study raise the possibility that reduced sleep duration may have a less severe impact than total sleep deprivation on factors important in cognitive therapy, such as emotional and cognitive processing. This finding is encouraging as it suggests that PTSD therapy can still be effective in the presence of reduced sleep duration, but does not preclude the possibility that sleep-focused treatment, before PTSD therapy could further enhance treatment gains. The finding that changes in sleep duration predicted posttreatment PTSD symptoms over and above initial symptom severity indicates greater treatment gains in patients whose sleep improved with treatment. A recent study found that sleep impairment improvements with sleep-directed hypnosis before PTSD therapy did not augment PTSD treatment gains (Galovski et al., 2016), however the effects of sleep treatments such as CBT for insomnia, have not yet been investigated, and may yield different results.

The present study also explored symptoms that may be associated with sleep improvements with CT-PTSD, by examining whether arousal, nightmares and pre-bed anxiety were affected by treatment, and whether changes were associated with sleep duration improvements (Question 5). The direction of effects between sleep improvement and improvements in nightmares, arousal and pre-bed anxiety were not determined in this study. However, the results, particularly differences in the effects of trauma and non-trauma-focused psychotherapy, generate some interesting hypotheses about how CT-PTSD may improve sleep duration, based on previously proposed mechanisms of therapeutic change with CT-PTSD (Ehlers et al., 2005).

There are several proposed mechanisms of PTSD symptom change with CT-PTSD (Ehlers et al., 2005). The first is a change in the trauma memory, through elaborating and updating the trauma memory with new meanings. In the current study, one possibility is that change in the trauma memory could have affected sleep duration through reducing both daytime and night-time intrusive memories and nightmares, and reducing hypervigilance and hyperarousal, and an overall sense of current threat. The results of the present study were consistent with this interpretation, as only CT-PTSD led to a greater reduction in nightmares than the waitlist, and nightmare and hyperarousal reduction with CT-PTSD were associated with improvements in sleep duration. For hyperarousal symptoms, supportive therapy also led to greater changes than the waitlist, possibly suggesting that a reduction in hyperarousal may be a less specific pathway to sleep duration improvements in patients with PTSD, than a reduction in nightmares. This would need to be explored in further research.

The second proposed pathway of PTSD symptom change with CT-PTSD is change in cognitive appraisals and related behaviours (Ehlers et al., 2005). Another possibility is that change in appraisals with CT-PTSD, including modifying appraisals of the need for vigilance, the safety of the bedroom and of the individual when they sleep, could theoretically increase sleep duration as a result of reduced pre-sleep hypervigilance, hyperarousal, and avoidance of the bed and sleep. The results of the present study may be consistent with this suggestion, as a reduction in anxiety about going to bed was only found with CT-PTSD, and not with supportive therapy, and was associated with sleep duration increases. However, the direction of effects between anxiety and sleep were not determined, and it is plausible that sleep duration improvements may lead to reduced anxiety about going to bed.

The finding that CT-PTSD but not supportive therapy led to greater improvements compared to the waitlist in anxiety about going to bed and nightmares, may help to explain why only CT-PTSD led to sleep duration and clinician assessed insomnia improvements compared to the waitlist, whereas both therapies led to greater PTSD symptom improvements than the waitlist. It is possible that improvements in these specific symptoms supported increase in sleep duration and reduction in insomnia symptoms.

More research is required to look at how reductions in re-experiencing, hyperarousal, and appraisal modification may relate to sleep improvements with CT-PTSD, and to determine the direction of effects, by exploring the temporal precedence of these PTSD symptom changes and their association with sleep improvements.

### 4.1 Limitations and future directions

The present study has a number of limitations. First, the sleep measure available regularly for all groups, at every time point, was self-reported average sleep duration for the previous week, rather than a validated self-report measure of sleep disturbances, or a measure assessing the broad range of sleep disturbances found in PTSD. However, sleep duration provided a useful indicator of sleep change with CT-PTSD, and improvements were also evidenced in sleep quality ratings and self-reported and clinician assessed insomnia symptoms and nightmares, suggesting that CT-PTSD also had beneficial effects on a broader range of sleep outcomes. Furthermore, objective measures of sleep duration were also not available. Some studies have shown that PTSD patients’ sleep diary reports of sleep duration are often consistent with actigraphy (Kobayashi, Huntley, Lavela, & Mellman, 2012), however it remains unknown whether CT-PTSD also has a beneficial effect on objectively measured sleep. Second, it is conceivable that the use of sleep diaries may have had a therapeutic effect on sleep symptoms in the intensive CT group, as daily self-monitoring of sleep before treatment may have a small beneficial effect on self-reported sleep (Talbot et al., 2014). However, this appears unlikely in the present study, as the standard CT group showed similar gains in sleep duration as the intensive CT group. Finally, as previously noted, memory updating was compared to no intervention rather than an alternative active intervention in CT-PTSD, which limits the conclusions that can be drawn from this finding. Further research should address these limitations, and investigate whether change in sleep with CT-PTSD is also reflected in objective data. Detailed sleep diaries meeting the recommendations for standardised prospective sleep self-monitoring (Carney et al., 2012) are also required, to assess variables such as sleep onset latency and wake time after sleep onset. Future studies could also further examine the mechanisms of improvements in sleep duration in trauma-focused CBT, and examine the specific effects of memory updating interventions in CT-PTSD by comparing memory updating to a different active intervention in CT-PTSD.

Overall, this study shows that self-reported sleep and insomnia symptoms improved with trauma-focused therapy for PTSD, and that sleep duration improved faster when PTSD treatment was delivered intensively over seven days. The results suggest that updating the trauma memory, a key procedure of CT-PTSD, was associated with sleep duration increase, although specific effects of memory updating were not determined. CT-PTSD may affect sleep duration through associated reductions in hyperarousal symptoms, nightmares, and pre-bed anxiety. Initial sleep duration did not predict PTSD treatment outcome, suggesting that CT-PTSD can be effective in the presence of reduced pre-treatment sleep duration. For many patients sleep difficulties were residual after PTSD treatment, raising the possibility that sleep treatment preceding or following PTSD treatment could enhance sleep gains and perhaps also PTSD treatment gains.

## References

[bib40] American Psychiatric Association (2013). Diagnostic and statistical manual of mental disorders.

[bib1] Belleville G., Guay S., Marchand A. (2011). Persistence of sleep disturbances following cognitive-behavior therapy for posttraumatic stress disorder. Journal of Psychosomatic Research.

[bib2] Blake D.D., Weathers F.W., Nagy L.M., Kaloupek D.G., Gusman F.D., Charney D.S. (1995). The development of a clinician administered PTSD scale. Journal of Traumatic Stress.

[bib3] Brownlow J.A., McLean C.P., Gehrman P.R., Harb G.C., Ross R.J., Foa E.B. (2016). Influence of sleep disturbance on global functioning after posttraumatic stress disorder treatment. Journal of Traumatic Stress.

[bib41] Buysse D.J., Ancoli-Israel S., Edinger J.D., Lichstein K.L., Morin C.M. (2006). Recommendations for a standard research assessment of insomnia. Sleep.

[bib4] Carney C.E., Buysse D.J., Ancoli-Israel S., Edinger J.D., Krystal A.D., Lichstein K.L. (2012). The consensus sleep diary: Standardizing prospective sleep self-monitoring. Sleep.

[bib42] Cox R.C., Olatunji B.O. (2016). A systematic review of sleep disturbance in anxiety and related disorders. Journal of Anxiety Disorders.

[bib6] Diekelmann S., Biggel S., Rasch B., Born J. (2012). Offline consolidation of memory varies with time in slow wave sleep and can be accelerated by cuing memory reactivations. Neurobiology of Learning and Memory.

[bib7] Ehlers A., Clark D.M. (2000). A cognitive model of posttraumatic stress disorder. Behaviour Research and Therapy.

[bib8] Ehlers A., Clark D.M., Hackmann A., McManus F., Fennell M. (2005). Cognitive therapy for post-traumatic stress disorder: Development and evaluation. Behaviour Research and Therapy.

[bib9] Ehlers A., Hackmann A., Grey N., Wild J., Liness S., Albert I. (2014). A randomized controlled trial of 7-day intensive and standard weekly cognitive therapy for PTSD and emotion-focused supportive therapy. American Journal of Psychiatry.

[bib43] First M.B., Gibbon M., Spitzer R.L., Williams J.B. (1996). User's guide for the structured clinical interview for DSM-IV axis I Disorders—Research version.

[bib10] Foa E.B., Cashman L., Jaycox L., Perry K. (1997). The validation of a self-report measure of posttraumatic stress disorder: The Posttraumatic diagnostic scale. Psychological Assessment.

[bib11] Galovski T.E., Harik J.M., Blain L.M., Elwood L., Gloth C., Fletcher T.D. (2016). Augmenting cognitive processing therapy to improve sleep impairment in PTSD: A randomized controlled trial. Journal of Consulting and Clinical Psychology.

[bib12] Galovski T.E., Monson C., Bruce S.E., Resick P.A. (2009). Does cognitive-behavioral therapy for PTSD improve perceived health and sleep impairment?. Journal of Traumatic Stress.

[bib13] Gutner C.A., Casement M.D., Gilbert K.S., Resick P.A. (2013). Change in sleep symptoms across cognitive processing therapy and prolonged exposure: A longitudinal perspective. Behaviour Research and Therapy.

[bib14] Hirshkowitz M., Whiton K., Albert S.M., Alessi C., Bruni O., DonCarlos L. (2015). National sleep Foundation's sleep time duration recommendations: Methodology and results summary. Sleep Health.

[bib16] Kleim B., Wilhelm F.H., Temp L., Margraf J., Wiederhold B.K., Rasch B. (2013). Sleep enhances exposure therapy. Psychological Medicine.

[bib17] Kobayashi I., Huntley E., Lavela J., Mellman T.A. (2012). Subjectively and objectively measured sleep with and without posttraumatic stress disorder and trauma exposure. Sleep.

[bib18] Kobayashi I., Mellman T.A., Altaee D., Howell M.K., Lavela J. (2016). Sleep and processing of trauma memories. Journal of Traumatic Stress.

[bib20] Levrier K., Marchand A., Belleville G., Dominic B.-P., Guay S. (2016). Nightmare frequency, nightmare distress and the efficiency of trauma-focused cognitive behavioral therapy for post-traumatic stress disorder. Archives of Trauma Research.

[bib21] Lommen M.J.J., Grey N., Clark D.M., Wild J., Stott R., Ehlers A. (2015). Sleep and treatment outcome in posttraumatic stress disorder: Results from an effectiveness study. Depression and Anxiety.

[bib23] Mitchell M.D., Gehrman P., Perlis M., Umscheid C.A. (2012). Comparative effectiveness of cognitive behavioral therapy for insomnia: A systematic review. BMC Family Practice.

[bib24] Nishith P., Duntley S.P., Domitrovich P.P., Uhles M.L., Cook B.J., Stein P.K. (2003). Effect of cognitive behavioral therapy on heart rate variability during REM sleep in female rape victims with PTSD. Journal of Traumatic Stress.

[bib25] Ohayon M.M., Shapiro C.M. (2000). Sleep disturbances and psychiatric disorders associated with posttraumatic stress disorder in the general population. Comprehensive Psychiatry.

[bib26] Pace-Schott E.F., Verga P.W., Bennett T.S., Spencer R.M.C. (2012). Sleep promotes consolidation and generalization of extinction learning in simulated exposure therapy for spider fear. Journal of Psychiatric Research.

[bib27] Raboni M.R., Tufik S., Suchecki D. (2006). Treatment of PTSD by eye movement desensitization reprocessing (EMDR) improves sleep quality, quality of life, and perception of stress. Annals of the New York Academy of Sciences.

[bib28] Schnyder U., Ehlers A., Elbert T., Foa E.B., Gersons B.P.R., Resick P.A. (2017). Psychotherapies for PTSD: What do they have in common?. European Journal of Psychotraumatology.

[bib44] Sinha S.S. (2016). Trauma-induced insomnia: A novel model for trauma and sleep research. Sleep Medicine Reviews.

[bib45] Talbot L.S., Maguen S., Metzler T.J., Schmitz M., Mccaslin S.E., Richards A. (2014). Cognitive behavioral therapy for insomnia in posttraumatic stress disorder: A randomized controlled trial. Sleep.

[bib34] Wagner U., Hallschmid M., Rasch B., Born J. (2006). Brief sleep after learning keeps emotional memories alive for years. Biological Psychiatry.

[bib35] Walker M.P., van der Helm E. (2009). Overnight therapy? The role of sleep in emotional brain processing. Psychological Bulletin.

[bib36] Woodward S.H., Arsenault N.J., Murray C., Bliwise D.L. (2000). Laboratory sleep correlates of nightmare complaint in PTSD inpatients. Biological Psychiatry.

[bib38] Yoo S.-S., Hu P.T., Gujar N., Jolesz F.A., Walker M.P. (2007). A deficit in the ability to form new human memories without sleep. Nature Neuroscience.

[bib39] Zayfert C., DeViva J.C. (2004). Residual insomnia following cognitive behavioral therapy for PTSD. Journal of Traumatic Stress.

